# Estimation of interaction energy and contact stiffness in atomic-scale sliding on a model sodium chloride surface in ethanol

**DOI:** 10.1038/s41598-018-22847-z

**Published:** 2018-03-16

**Authors:** Liron Agmon, Itai Shahar, Danny Yosufov, Carlos Pimentel, Carlos M. Pina, Enrico Gnecco, Ronen Berkovich

**Affiliations:** 1 Department of Chemical Engineering, Ben-Gurion University of the Negev, Beer Sheva, 8410501 Israel; 2 Departamento de Cristalografía y Mineralogía, Universidad Complutense de Madrid, E-28040 Madrid, Spain; 3 Instituto de Geociencias, E-28040 Madrid, Spain; 4 Otto Schott Institute of Materials Research, Friedrich Schiller University Jena, D-07742 Jena, Germany; 5 The Ilse Katz Institute for Nanoscale Science and Technology, Ben-Gurion University of the Negev, Beer Sheva, 8410501 Israel

## Abstract

Friction force microscopy (FFM) in aqueous environments has recently proven to be a very effective method for lattice-resolution imaging of crystal surfaces. Here we demonstrate the use of ethanol for similar measurements on water-soluble materials. Lattice resolved frictional stick-slip traces of a cleaved NaCl(100) surface submerged in ethanol are compared with previous obtained FFM results in ultrahigh vacuum (UHV). We use the Prandtl-Tomlinson framework to estimate the amplitude of the corrugation potential and the contact stiffness. The surface potential amplitude scales with the applied normal loads are in good agreement with data obtained for NaCl measured under UHV conditions, but demonstrates deviations from the ideal periodic potential given by the Prandtl-Tomlinson model. An additional finding is that the use of ethanol allows us to explore higher load ranges without detectable evidence of surface wear. The contact stiffness does not vary significantly with the normal load up to 38 nN, while above it a sudden increase by almost one order of magnitude was observed. Comparing this to previous results suggests that considerable atom rearrangements may occur in the contact region, although the (100) surface structure is preserved by ethanol-assisted diffusion of Na and Cl ions.

## Introduction

With the advent of friction force microscopy (FFM) as a variant technique of atomic force microscopy (AFM), it became possible to disclose in the sub-nanometer scale information on the underlying tribological mechanisms, on crystal lattices and sometimes even on atomic structures^1–5^. With the use of AFM apparatus, FFM experiments record atomic-scale forces via the interaction of an ultra-sharp cantilever tip with the surface of interest, while probing it by scanning under an applied normal load. This enables a direct measurement of lateral friction forces that exhibit atomic stick-slip pattern as the tip slides across a crystal substrate. Up to recently, most of FFM experiments showing lattice resolution were performed under ultrahigh-vacuum (UHV) or in other environments (for instance in N_2_ or Ar)^1,6–11^, which circumvent complications that may arise due to capillary forces and possible contaminations^12,13^. This concern lately met an alternative in the form of performing FFM experiments in liquid surroundings^14–19^, although high resolution measurements in liquid environment were shown more than a decade earlier^20^.

In light of this increasing trend, Vilhena *et al*.^19^ addressed the question of the validity of FFM experiments in liquid environment, joining FFM experiments with molecular dynamic (MD) simulations of graphene in water and under UHV conditions. They concluded that the remarkable lattice resolution reported when performing FFM experiments in liquid is not incidental, and thus can be regarded as a highly practical methodology. This statement was based on their founding that the liquid molecules do not impair the spatial resolution of the measurement, but merely introduce a stochastic effect that result in a slightly noisier recording. Here we continue this line of study by performing FFM measurements on NaCl immersed in ethanol, and comparing these measurements with FFM measurements in UHV reported by Socoliuc *et al*.^9^.

The mineral halite (NaCl) has perfect cleavage along {100} planes, which show atomically well-defined surface widely explored in nanotribology, and particularly in FFM under UHV conditions^1,9,13,21,22^, making this natural compound a substance well suited for further exploration in liquid surroundings. We use ethanol as submersing medium due to the low solubility of NaCl in it (0.00065 g_NaCl_/g_ethanol_). For comparison, NaCl solubility in water is about three orders of magnitude higher (0.36 g_NaCl_/g_water_)^23^. The extremely high solubility of NaCl in water results in the rapid retreat of their surfaces, making virtually impossible high resolution imaging in aqueous environment. In contrast, we achieved lattice resolution of the NaCl {100} faces through FFM measurements in ethanol covering a wide dynamical scanning range (1–110 nm/s) under normal loads ranging between 2.3–58.4 nN. Observing velocity strengthening of the recorded normal force, we estimated the amplitude of the corrugation effective potential between the substrate and the AFM probe using the *Prandtl*-*Tomlinson* (PT)^24,25^ model at an activation temperature. We show that our measurements performed in liquid environment agree and scale with FFM measurements of NaCl under UHV conditions.

## Results and Discussion

**Figure 1:**
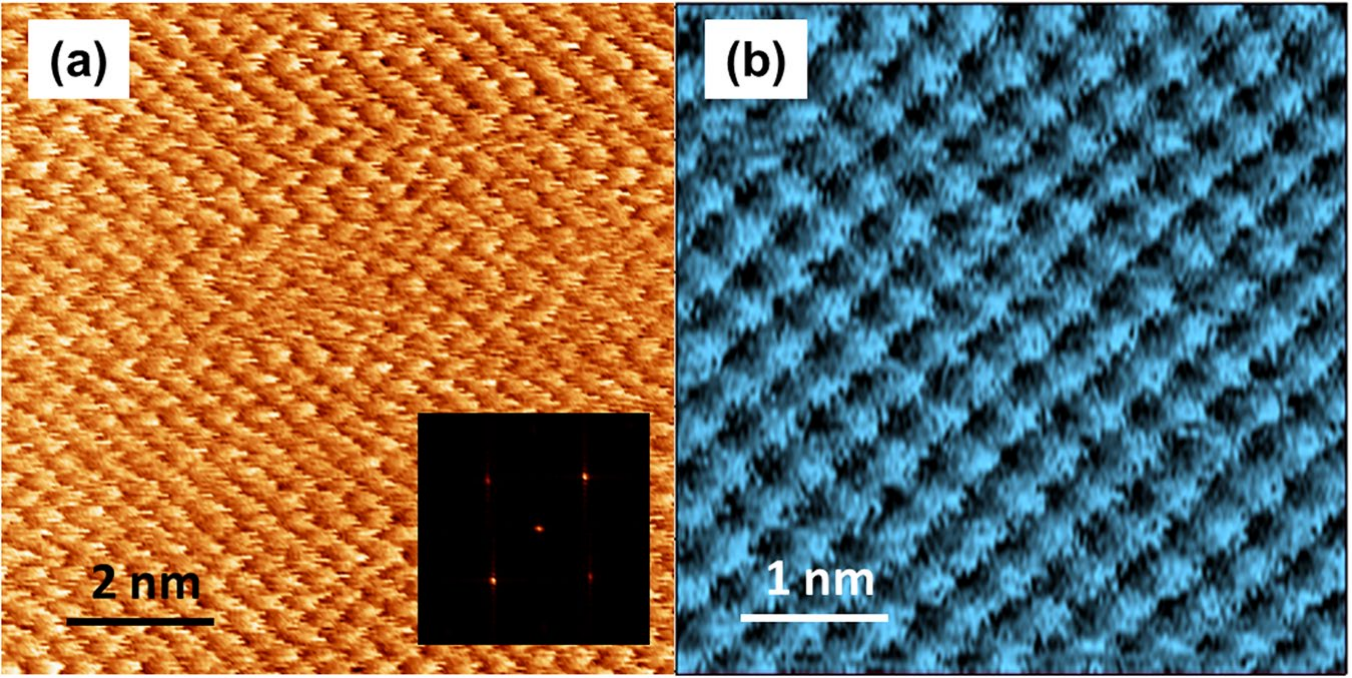
Lateral friction force maps of NaCl showing the atomic lattice structure the surface of NaCl(100) immersed in ethanol. (**a**) 10 × 10 nm^2^ map measured with Setup I, under an external normal load *F*_*N*_ = 12.1 nN and 2D FFT (inset). (**b**) 5 × 5 nm^2^ map measured with Setup II, under an external normal load *F*_*N*_ = 11 nN.

Friction force microscopy experiments on NaCl immersed in ethanol showed the characteristic stick-slip pattern that result in lattice resolution. To demonstrate the generality of measuring the NaCl in ethanol, Fig. 1 shows two exemplary lateral friction force maps measured using two different commercial AFMs, referenced as Setup I and Setup II (see methods section for details). Figure 1a shows one of the first high-resolution friction images of NaCl immersed in ethanol recorded using Setup I. This image demonstrates the feasibility of conducting detailed nanotribological studies on NaCl surfaces in liquid. Applying two dimensional Fast Fourier transform (2D-FFT) to the friction map (inset of Fig. 1a) we measured a lattice constant of about 0.59 nm. Measurements on subsequent images recorded with Setup II (Fig. 1b) provided a distance between slip peak events of 0.584 ± 0.05 nm. Both measured lattice constants are in close proximity with the lattice periodicity of NaCl (*a* = 0.564 nm) obtained from diffraction data, indicating that NaCl surfaces in ethanol do not experience any significant structural relaxation. Note that the remarkable lattice resolution shown in the friction maps, measuring sequences of individual friction loops, could not be obtained when the measurements were performed with similar parameter values in air. Compared to FFM measurements in UHV, operation in ethanol enabled exploring a wider dynamical range of normal forces and scanning velocities. FFM measurements are more stable with Setup II due to its temperature controller and electronics (as it dramatically reduces artifacts inherent to a piezoelectric scanner using a closed loop capacity). For this reason, we will focus on such nanotribological measurements with this setup in the following.

**Figure 2:**
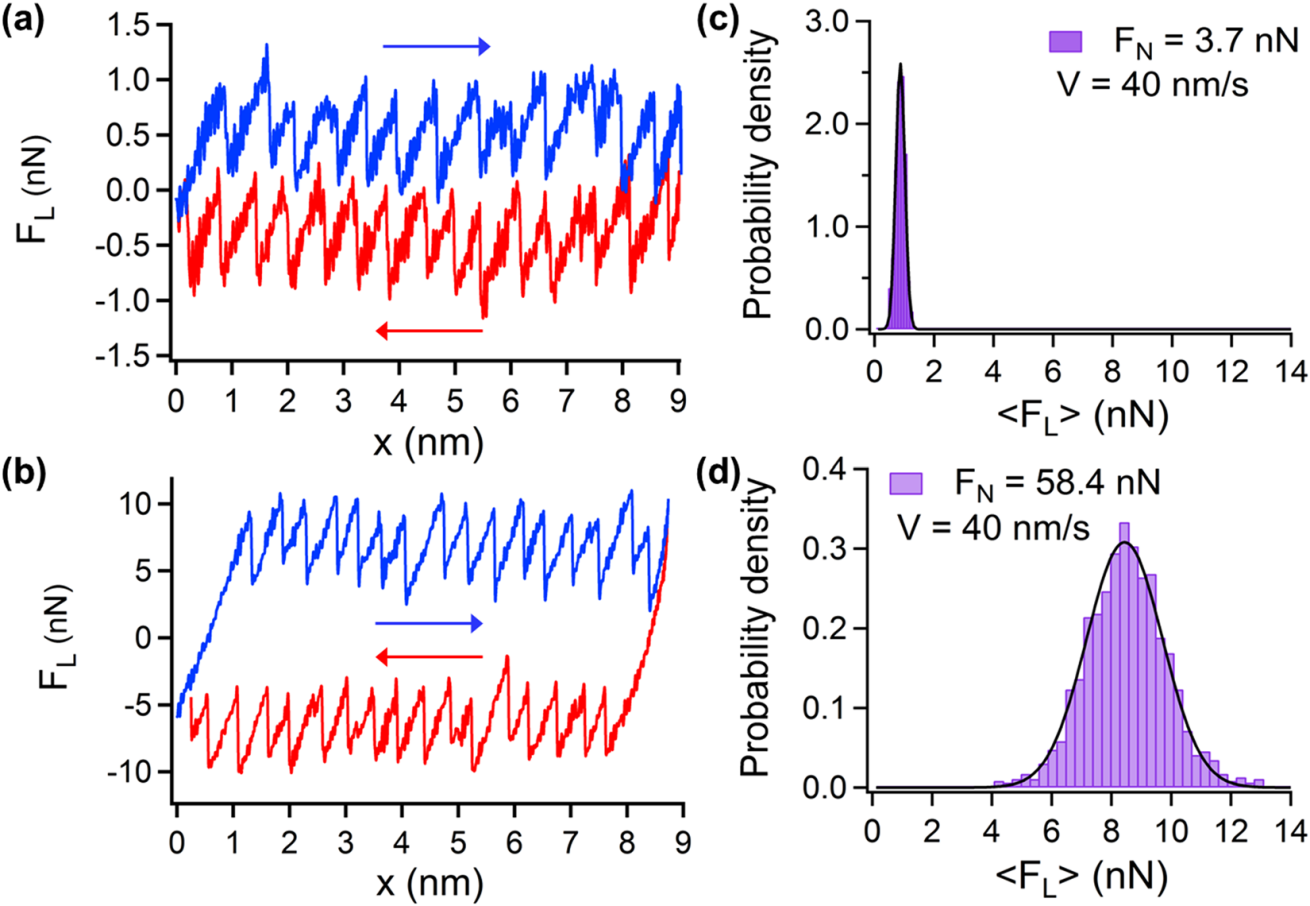
FFM stick slip measurements of NaCl in ethanol. (**a**) Friction loop obtained at a scanning velocity of 40 nm/s under normal loads *F*_*N*_ = 3.7 nN and, (**b**) *F*_*N*_ = 58.4 nN. The forward and backward traces of lateral forces are marked in blue and red, respectively and the scanning directions are also indicated by arrows with the same colors. (**c**) and (**d**) show the probability density functions of the lateral friction forces measured at the same experimental conditions shown in (**a**) and (**b**), respectively.

Figure 2 shows two exemplary traces of lateral force loops obtained under extreme values for the normal forces, *F*_*N*_ = 3.7 and 58.4 nN, at a scanning velocity of 40 nm/s. The hysteresis between the forward (blue) and backward (red) curves [traces] strongly increases with the normal load as can be seen when comparing the 3.7 nN friction loop (Fig. 2a) to the 58.4 nN friction loop (Fig. 2b). The increase of normal load corresponds to more energy dissipation. This trend was previously demonstrated on NaCl under UHV conditions albeit over a smaller range of normal loads (0.47 to −4.7 nN)^9^. The probability density functions (pdf) of the lateral forces calculated from traces measured under 3.7 nN and 58.4 nN are shown in Fig. 2c,d, respectively. The effect of the normal load can be quantified, as the mean lateral force under the low load of 3.7 nN, estimated with a Gaussian fit^8^, <*F*_*L*_> 0.80 ± 0.15 nN (*N* = 397) at *F*_*N*_ = 3.7 n*N* grows under the high load of 58.4 nN to <*F*_*L*_> = 8.30 ± 1.27 nN (*N* = 1538) at *F*_*N*_ = 58.4 nN.

**Figure 3:**
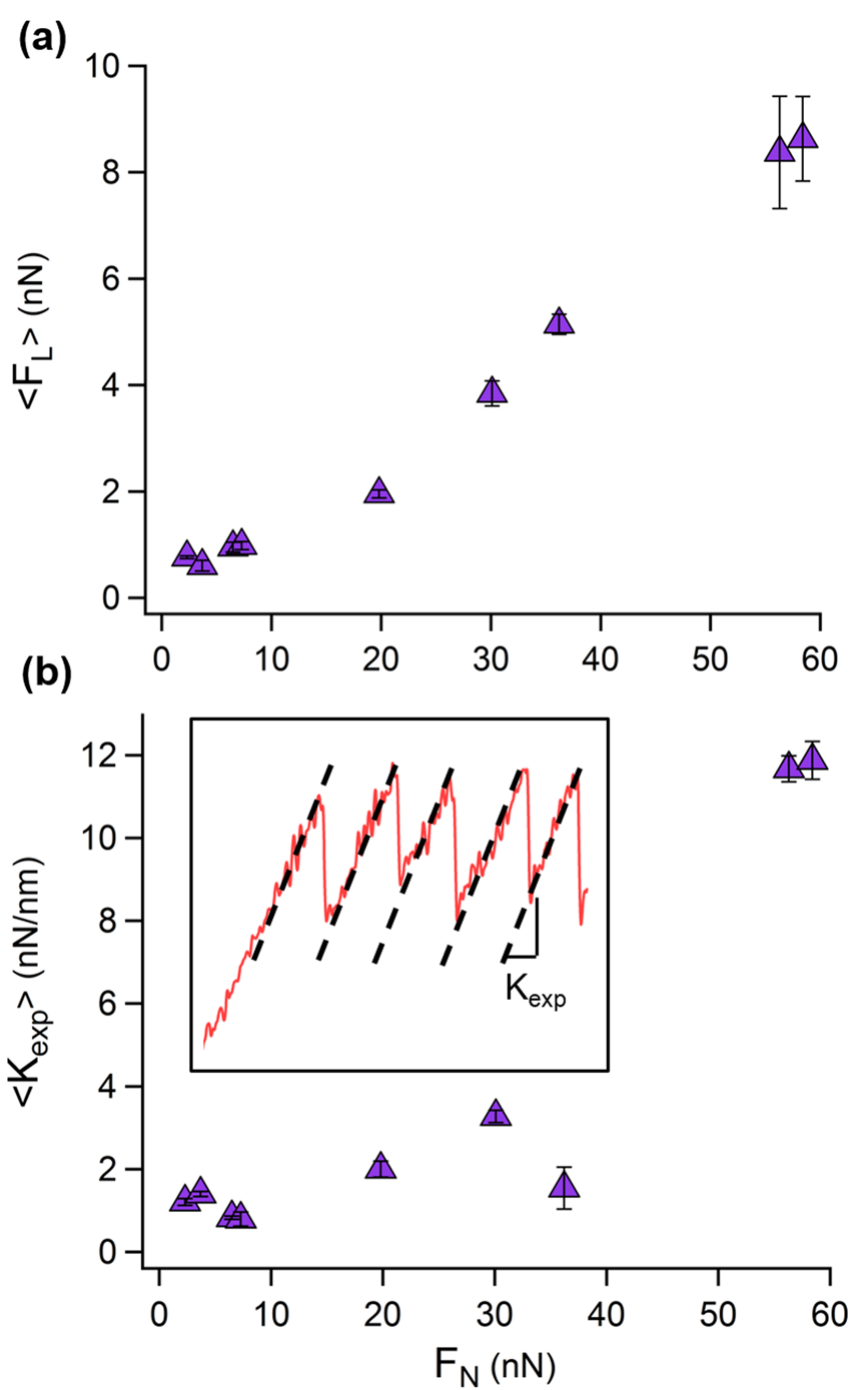
Normal load dependencies from FFM measurements of NaCl in ethanol. (**a**) Mean lateral friction force dependency on the applied normal force. (**b**) Dependency of the mean experimental contact stiffness, <*K*_exp_>, on the normal force, measured from the slope of the stick phase (inset).

From the measured friction loops displaying stick-slip we calculated the mean lateral forces, <*F*_*L*_>, by averaging their maximal slip values at each applied normal load, and taking their standard deviations in their averaging as the errors. Plotting the mean lateral force values against the applied normal loads in Fig. 3a shows an increase of the lateral force with the normal load, with a slope that defines the friction coefficient of *μ* = 0.146 ± 0.007. This value is larger than the one reported for NaCl when probed with Ag/Si tip in UHV, *μ* = 0.01 − 0.04^26^ consistent with the well-known fact that liquids on sliding surfaces of minerals considerably increase the frictional coefficients of crystal surfaces^27^. The contact stiffness between the probe and the NaCl sample was estimated by taking the slope of the recorded force (with respect to the support displacement) in the stick phase (*K*_exp_ = *dF*_*L*_/*dx*) of the lateral force loop^28^. These slopes were averaged for each normal load to provide the measured mean contact stiffness values, <*K*_exp_>, which are shown in Fig. 3b. The contact stiffness appears to moderately increase up to ~40 nN, however, at higher values it has dramatically increased by one order of magnitude. Interestingly, at loads above 30 nN, the distributions of the lateral forces become broader, as indicated by their standard deviations in Fig. 3a. This increase of the lateral force scattering at high loads and the corresponding increase of the contact stiffness can be attributed to a sudden increase of the contact area when the external pressure exceeds a certain threshold. In this case, the NaCl surface is locally damaged by the tip, although ethanol-assisted diffusion of Na and Cl ions may cause a prompt recovery of the structure of {100} surfaces. Another interesting aspect of measuring in liquid surroundings is the possibility to apply high normal loads (such as 58 nN) while still recovering a clear stick-slip pattern with lattice resolution. It is possible that several ethanol molecules get in the gap between the tip and the substrate providing a minor lubricating effect or that the ethanol here interacts with the NaCl surface and prevents wear. Nevertheless, the origin of this remain unclear.

We interpret our measurements within the framework of the PT model, which depicts the tip-sample dynamical interaction as a mass point that excurses a potential. A convolution between a harmonic term (the cantilever tip), *U*_*h*_ = (*K*_*eff*_/2)(*x*_*t*_ − *X*_*s*_)^2^ and a periodic term (the probed surface), *U*_*p*_ = −*U*_0_cos(2*πx*_*t*_/*a*) results in an effective interaction potential, *U*(*x*_*t*_,*t*) = *U*_*p*_(*x*_*t*_) + *U*_*h*_(*x*_*t*_,*t*). The harmonic term describes the position of the AFM cantilever tip at time *t*, *x*_*t*_(*t*), dragged by the support, *X*_*s*_ = *Vt*, by means of an effective torsional elastic spring constant, *K*_*eff*_, accounting for the collective effect of the elastic deformation of the tip and the interaction region^28,29^. The periodic term is characterized by *U*_0_, the unperturbed corrugation amplitude, with a lattice periodicity *a*. According to this description, the lateral force is stated by *F*_*L*_ = −*K*_*eff*_(*x*_*t*_ − *Vt*). Nevertheless, one has to bear in mind that this description is a simplification, since the motion of the tip is two dimensional in practice^30,31^. A dimensionless number, which may be called the PT parameter^9,32,33^, defines the relation between the corrugation amplitude and the elastic energy:

1 $$\eta ={(\frac{2\pi }{a})}^{2}\frac{{U}_{0}}{{K}_{eff}}$$

and was shown to determine the effective lateral stiffness by correcting the measured experimental stiffness through^9,32^

2 $${K}_{eff}=(\frac{\eta +1}{\eta }){K}_{\exp }.$$

When *η* >> 1, then *K*_*eff*_ ~ *K*_*exp*_^28^, however when *η* approaches 1, then this correction is required^32^, while for *η* < 1, stick slip conditions are typically not met^9^.

Reproduction of the mean corrugation potential amplitude for every normal load was achieved using the thermally activated PT model (PTT) that predicts the following relationship between the mean lateral forces and the scanning velocity^2,5,34–37^:

3 $$\begin{array}{c}\langle {F}_{L}(V,T)\rangle ={F}^{\ast }-{[\varphi {k}_{B}T\mathrm{ln}(\frac{{V}_{C}}{V})]}^{\frac{2}{3}};\\ {V}_{C}=\frac{2{f}_{0}\varphi {k}_{B}T}{3{K}_{eff}\sqrt{{F}^{\ast }}}\end{array}$$

where *f*_0_ is the characteristic attempt frequency resulting from the oscillations of the cantilever apex within the potential well, *k*_*B*_ is Boltzmann’s constant, *T* is the absolute temperature, F* is the extrapolation to the normal force dependent slip force at zero temperature. The curvature of the underlying potential is parameterized by *ϕ*, which assumes the following form for a periodic potential according to the PT model: *ϕ* = *δϕ*^*PT*^, where *ϕ*^*PT*^ = (3*π*/*a*)(F*/8)^1/2 ^^35^. *δ* is a factor that was introduced to enable deviations from the perfectly sinusoidal potential assumed in the PT model, which may not necessarily represent adequately the actual curvature of the potential probed experimentally^37^. *K*_*eff*_ is estimated via equation (2), when the PT parameter can also be estimated from the experimental data using the mean lateral forces and the contact stiffness according to *η* = *η*_*exp*_ = 2*π* < *F*_*L*_ >/(*K*_*exp*_*a*) − 1^9^.

**Figure 4:**
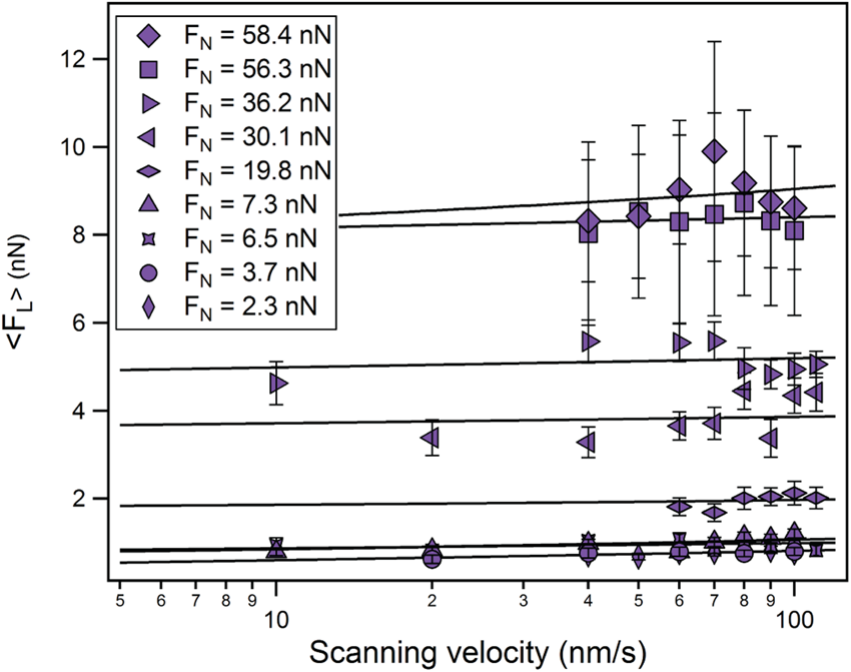
Mean lateral force at different applied normal loads (symbols) as a function of scanning velocity, fitted with the PTT model given by equation (3) (solid lines).

Figure 4 shows the averaged maximum lateral force-velocity dependency, for all the applied normal forces. While the friction increased notably as a function of the normal load, we observed only a moderate strengthening of the mean lateral forces with scanning velocity. Since we did not encounter plateauing of the lateral forces at high scanning velocities^35,38^, we fitted our data with the low velocity relationship given in equation (3) for fixed values of *f*_0_ ranging from 3–100 MHz to achieve adequate convergence (straight lines on Fig. 4).

**Figure 5:**
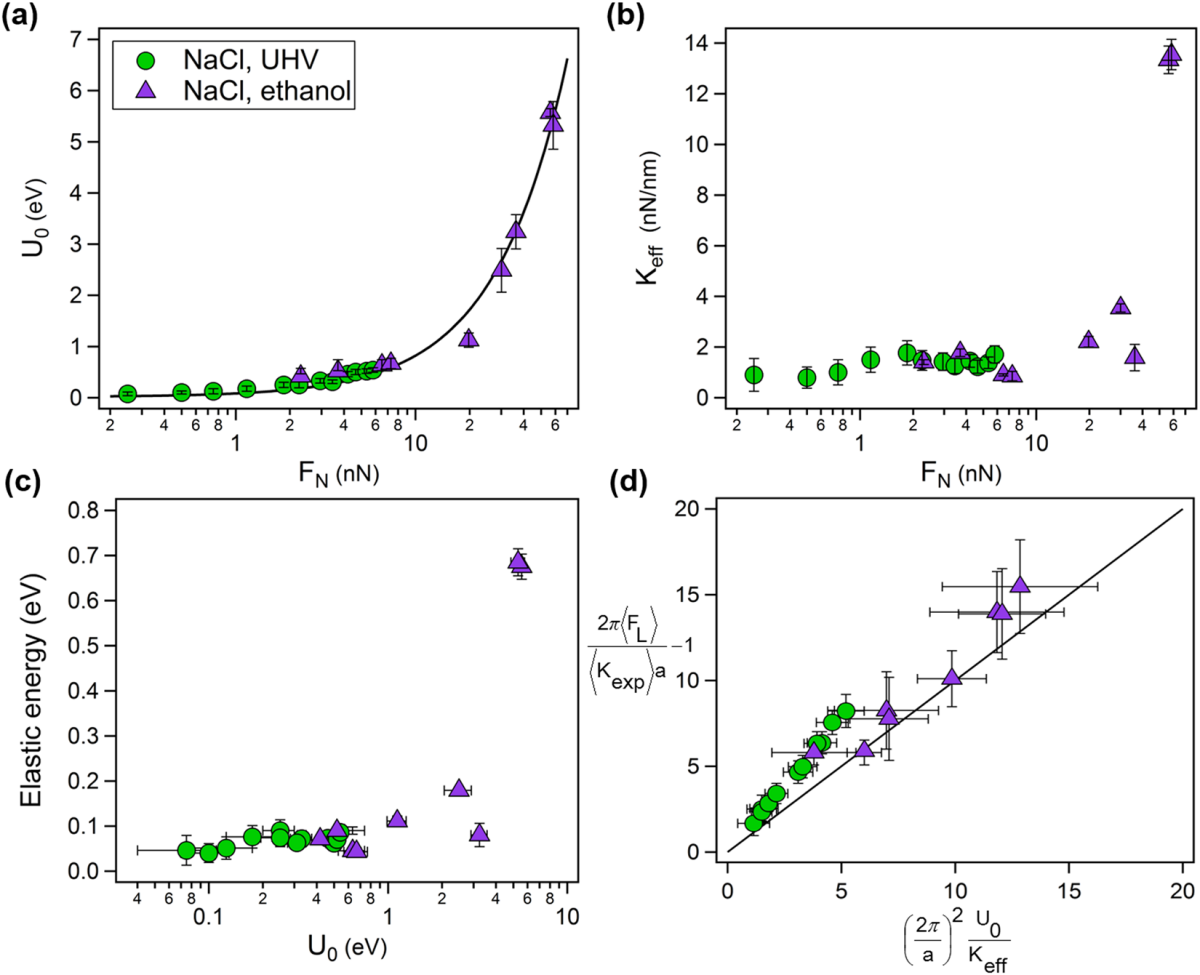
NaCl in ethanol (purple triangles) compared with NaCl in UHV (green circles, data adapted form ref.^9^ with the kind permission of Prof. E. Meyer). (**a**) Amplitude of the corrugation potential, *U*_0_, as a function of the normal load. (**b**) Effective lateral stiffness *k*_*eff*_ dependency with the normal load. (**c**) Relation between the elastic energy of the contact with the amplitude to the corrugation amplitude. (**d**) Variation between the experimental and analytical PT parameter.

The extrapolated zero temperature force, F*, increased with the normal load from 0.74 nN to 9.89 nN while *δ* and *f*_0_ did not exhibit any discernible trend, but fluctuated according to the scattering of the data. At this point, it is difficult to determine whether the fitted value of *δ* = 4.83 ± 2.36 is indicative of deviation of the data from the ideal curvature of the sinusoidal potential given by the PT model, or rather due to the scattering of some of the data sets per given normal load. F* can be used in a first order approximation of a local barrier that is being crossed. This leads to the definition of the corrugation amplitude of the potential as *U*_0_ ≈ *F* *a/(2*π*)^9^, which was estimated for every applied normal load. These values were compared with corrugation energy values measured for NaCl under UHV conditions^9^, and are shown in Fig. 5a. Interestingly, both of the data sets remarkably scale with an overlap in *U*_0_ in the low load region (below 10 nN). We propose the following ansatz to describe the combined results:

4 $${U}_{0}=\varepsilon {(\frac{{F}_{N}-{F}_{0}}{{F}_{0}})}^{n}$$

with *n* being a general power, *ε* ≡ *U*_0_|_*FN* = 0_ is the amplitude of the corrugation interaction potential at zero force, *F*_0_ represents the interaction force when *U*_0_ = 0 under which no interaction with the surface exists and implies that the intramolecular forces acting between the tip and the surface become attractive. The value of *F*_0_ can be considered as an adhesion force, such as the ones experiences when approaching with the tip to the surface (see Fig. 3 in ref.^19^), or formation of necking between the tip and the surface^39^. This model empirically describes the combined data obtained from FFM measurements in UHV and in ethanol reasonably well, with the following values: *n* = 1.084, *ε* = 0.014 eV and *F*_0_ = 0.245 nN, suggesting that the interaction amplitude increases linearly with the applied normal load. Interestingly, similar behavior of the surface potential (by means of the surface curvature) with the applied load at frictional interfaces was demonstrated using *ab-initio* calculations, while employing the PT model^40^.

In agreement with Socoliuc *et al*.^9^, under loads smaller than 10 nN, the effective contact stiffness remains relatively constant, fluctuating around 1 N/m (Fig. 5b). However, at higher loads, *K*_*eff*_ increases as well, in a similar fashion as *K*_*exp*_ shown in Fig. 3b. The increase of the corrugation amplitude of the potential with the applied load corresponds to the increase of the effective contact stiffness. This consistency in the increase of *U*_0_ and *K*_*eff*_ with the applied load indicates that changes occur within contact area, as the barrier height between the atoms involved in the contact area becomes deeper. Under loads higher than 10 nN the structure at the interface between the NaCl surfaces and the apex of the tip considerably deforms, as the tip indents deeper into the surface lattice. Figure 5c shows the interplay between the elastic energy of the contact, given by *K*_*eff*_[*a*/(2*π*)]^2^, and the corrugation energy, *U*_0_. Dominated by the contact stiffness, the elastic energy persists around 0.1 eV within the range of up to 1 eV in the corrugation energy. Beyond this value, the elastic energy grows rapidly to 0.68 eV, as the corrugation energy grows to 5.8 eV. Hence, there is a strong correlation between the increases of elasticity of the contact region with the deepening of the corrugation potential, which becomes prominent as the applied normal load is raised. Figure 5d plots the PT parameter, *η*_*exp*_, calculated directly from the measured data (in UHV and ethanol) versus the analytical formulation of the PT parameter, given by equation (1), which was calculated using the parameters obtained after processing the data (measurements in ethanol). UHV values were taken from ref.^9^. The two PT parameters do not collapse over the main linear master curve with a slope of unity that tangentially crosses the figure. The UHV data at low values of the PT parameter relatively match each other, and deviate from each other as it grows, inclining towards the PT parameter that was directly calculated from the experimental data. The data measured in ethanol is more scattered, however, it still displays similar trend at higher values of the PT model, although to much less extent (considering the large error bars). This further adds to the fitted values of *δ* in equation (3) that are attributed to an apparent deviation of the measured mean potential curvature from the ideal sine given in the PT model^37^. Indeed, if *δ* would be close to 1, then the actual effective interaction potential could be described by a perfect sine, and one would expect that all the data point would fall onto the linear master curve. Although *δ* did not exhibit any correlation with the applied load, the tendency of separation of the PT parameter from ideality (i.e., from a sine shape) indicates that at *η*_*exp*_/*η* ~ 1 the PT model serves as a relative good approximation for the contour of the surface corrugation potential, while as *η*_*exp*_ increases, the curvature departs from this description. Such deviations from ideality in the surface potential can be explicitly explored using MD simulations^19^ and *ab-initio* calculations^40–42^, which can additionally provide detailed insights on effect of the solvent molecules in the interface^43^.

## Conclusion

Nanotribological experiments have been performed on NaCl crystals immersed in ethanol, a solvent that maintained the intactness of the surface, and enabled achieving high-resolution stick-slip friction loops. The mean lateral forces of the reported measurements showed force strengthening with the scanning velocity, and were analyzed within the framework of the PTT model. The calculated amplitude of the corrugation energies and effective contact stiffness showed that they scaled with similar parameters reported of NaCl in UHV^9^. Furthermore, we showed that the potential corrugation surfaces might exhibit non-homogenous and non-uniform potential contour that deviates from the ideal description posed by the PT model Most importantly, this study provides another step in establishing the methodology of performing FFM experiments in liquid surroundings.

## Methods

Halite (NaCl) crystals were freshly cleaved along the {100} planes and submersed in ethanol 99.9% (Romical). Friction AFM images of halite {100} surfaces in ethanol were first obtained using the Nanoscope IIIa Multimode AFM (Veeco Instruments) at Complutense University, Spain (Setup I) with Bruker SNL-10D Silicon-nitride cantilevers with Si-tips, with a normal spring constant of *K*_*N*_ = 0.06 N/m, inverse lever sensitivity of 59.72 nm/V and a lateral spring constant of *K*_*T*_ = 33.35 N/m that was calculated following Noy’s method^44^. Other friction maps and friction-loops (that were used for the data analysis in this paper) were obtained with Asylum Research Cypher-ES AFM (Oxford Instruments), at Ben-Gurion University, Israel (Setup II). The calibration of lateral forces, measured in volts, was done using the wedge method^45–47^, using Bruker SNL-10D Silicon-nitride cantilevers with Si-tips, with a normal spring constant of *K*_*N*_ = 0.05 ± 0.01 N/m, inverse lever sensitivity of 47.07 ± 3.43 nm/V and the wedge conversion factor (lateral sensitivity), *α* = 135.4 ± 27.9 nN/V (corresponding to lateral spring constant of *K*_*T*_ = 38.95 ± 5.55 N/m). Friction traces (loops) of the NaCl surface were recorded with low integral gain (0.01). We scanned back and forth 5–40 nm under a 90° scan angle, recording single stick-slip events on the NaCl surface while varying the scan rate after acquiring at least 250 cycles per scanning velocity. Both AFMs were operated in contact (lateral friction microscopy) mode and the measurements were conducted at room temperature.
